# Defining and evaluating the Hawthorne effect in primary care, a systematic review and meta-analysis

**DOI:** 10.3389/fmed.2022.1033486

**Published:** 2022-11-08

**Authors:** Christophe Berkhout, Ornella Berbra, Jonathan Favre, Claire Collins, Matthieu Calafiore, Lieve Peremans, Paul Van Royen

**Affiliations:** ^1^Department of General Practice/Family Medicine, Université de Lille, Lille, France; ^2^Department of Family Medicine and Population Health, Universiteit Antwerpen, Antwerp, Belgium; ^3^Irish College of General Practitioners, Dublin, Ireland; ^4^ULR 2694 METRICS, Université de Lille, Lille, France; ^5^Department of Nursing and Midwifery, Universiteit Antwerpen, Antwerp, Belgium

**Keywords:** effect modifier/epidemiologic, scientific experimental error, systematic review, primary healthcare, Hawthorne effect

## Abstract

In 2015, we conducted a randomized controlled trial (RCT) in primary care to evaluate if posters and pamphlets dispensed in general practice waiting rooms enhanced vaccination uptake for seasonal influenza. Unexpectedly, vaccination uptake rose in both arms of the RCT whereas public health data indicated a decrease. We wondered if the design of the trial had led to a Hawthorne effect (HE). Searching the literature, we noticed that the definition of the HE was unclear if stated. Our objectives were to refine a definition of the HE for primary care, to evaluate its size, and to draw consequences for primary care research. We designed a Preferred Reporting Items for Systematic reviews and Meta-Analyses review and meta-analysis between January 2012 and March 2022. We included original reports defining the HE and reports measuring it without setting limitations. Definitions of the HE were collected and summarized. Main published outcomes were extracted and measures were analyzed to evaluate odds ratios (ORs) in primary care. The search led to 180 records, reduced on review to 74 for definition and 15 for quantification. Our definition of HE is “an aware or unconscious complex behavior change in a study environment, related to the complex interaction of four biases affecting the study subjects and investigators: selection bias, commitment and congruence bias, conformity and social desirability bias and observation and measurement bias.” Its size varies in time and depends on the education and professional position of the investigators and subjects, the study environment, and the outcome. There are overlap areas between the HE, placebo effect, and regression to the mean. In binary outcomes, the overall OR of the HE computed in primary care was 1.41 (95% CI: [1.13; 1.75]; *I*^2^ = 97%), but the significance of the HE disappears in well-designed studies. We conclude that the HE results from a complex system of interacting phenomena and appears to some degree in all experimental research, but its size can considerably be reduced by refining study designs.

## Introduction

By autumn of every year, the main French mandatory health insurance scheme conducts a promotional campaign for seasonal influenza vaccination in mass media and in health facilities. General practice surgeries can participate in this campaign by hanging posters and making pamphlets available in their waiting rooms. Advertising using posters and pamphlets in waiting rooms shows no evidence of effectiveness in terms of increasing knowledge or changing the health behavior of patients (1). We conducted a cluster-randomized controlled trial (RCT) with 10,597 patients assessing the 2014–2015 campaign in France confirming these findings (2). No difference was demonstrated in vaccination uptake between waiting rooms advertising for influenza vaccination (intervention) or not (controls) (*P* = 0.561). However, the immunization rate increased by about 3% in both arms of the trial compared to the baseline (previous year). At the same time, a decrease in coverage of 2.4% was observed district wide by public health authorities. As our trial targeted a change in behavior in primary healthcare, we considered the possibility of a Hawthorne effect (HE) to explain this difference and felt the need to have greater insight regarding this effect (3).

The Hawthorne effect (HE) was first observed in relation to six, partly overlapping, experiments carried out from 1924 to 1933 at the Hawthorne plant, a large factory complex of the Western Electric Company in Cicero (Illinois, USA), also reputed to have generated Al Capone’s original fortune (4). The most thorough publication was issued by Roethlisberger and Dickson which presented data from the six experiments (5). Elton Mayo, a Harward business professor, was not the director of the studies, but as he became the main interpreter of the Hawthorne experiments, his name remains associated with the research (6). The study group examined the effects of various incentives on the productivity of two groups of volunteer workers, and the good story was that whatever experiment was applied, the trend of productivity was upward in both groups (7). However, this does not fit with the two last experiments (6). The term “Hawthorne effect” or “observer effects” to describe the performance or behavior improvement of people involved in research, arising exclusively when under observation, was first used in 1953 (8). In 1974, Parsons described the HE as a failure of the experimenters to realize how the consequences of subjects’ performance affect what subjects do (9). Indeed, the internal validity of the Hawthorn experiments was biased by the selection of a small number of volunteer participants, attrition due to the removal of operators because of gross insubordination, and potential antagonism between management and employees (Dickson was an officer of the Western Electric Company) (6). In 2011, Levitt and List recovered the original results of the Hawthorne illumination experiments and reanalyzed the outcomes, finding “some weak evidence that workers respond more to experimental manipulations than to naturally occurring changes in light (10).”

In 2010, French and Sutton published a narrative review calling the changes in the people being measured in an experimental environment “measurement reactivity.” They merged this designation with other terms including “assessment reactivity,” “mere measurement,” “question-behavior effect,” or “self-generated validity” (11). Further, in 2017 Paradis and Sutkin recommended the use of the phrase “participant reactivity” when considering the triad participant, observer, and research question (12). One common point of all effects appearing in an experimental environment, whatever their designation, is the considerable heterogeneity of their size across studies (13, 14).

In 2014, McCambridge et al. published an often-cited systematic review to elucidate the existence of the HE, the conditions of its appearance, and its estimated size (15). They noted that it was relevant to clear the term HE in health sciences, as it was evoked in relation to a range of methodological phenomena. To define the HE, they stated that “awareness of being observed or having behavior assessed engenders beliefs about researcher expectations. Conformity and social desirability considerations then lead behavior to change in line with these expectations.” They came to the conclusion that “Further research on this subject should be a priority for the health sciences, in which we might expect change induced by research participation to be in the direction of better health and thus likely to be confounded with the outcomes being studied (15)”.

In 2020, Purssell et al. conducted a systematic review and meta-analysis regarding the HE in hand hygiene (HH), based on the many publications in the field related to the guidance for HH promoted by the World Health Organization (WHO) (“My Five Moments for Hand Hygiene” initiative) in 2009 (16). It confirmed the considerable heterogeneity in outcomes, with the HE ranging from -6.9 to 65.3%. Probably in line with this heterogeneity, they did not complete the meta-analysis (17). Hand-hygiene behaviors have markedly changed since the COVID-19 outbreak (18). For this reason, the outcomes regarding hand hygiene in hospital wards as in the community are probably outdated.

Noting the considerable inconsistency regarding the phenomenon, the primary objectives of this review were (1) to refine the definition of the HE and outline the progress of research since 2012 (last inclusions in McCambridge’s review) on the HE in terms of its existence and characteristics and (2) to estimate its size in primary care studies, expecting the already described heterogeneity.

## Materials and methods

### Eligibility criteria, information sources, and search strategy

Considering the definition, publications related to research in the medical field, in particular those regarding health professionals and patients, were included. Reports needed to contain a clear definition or outcome measuring the HE. Included methodologies were clinical trials and their reanalysis, quasi-experimental or observational studies, or historical comparisons. Reports published in French or English, with an available abstract, were included. Only reports published after the review by McCambridge were considered (publication range: January 2012 to March 2022). We ensured that no reports were overlapping with McCambridge’s review (15).

Reports outside the field of medicine or human behavior related to health and those citing the HE without definition or outcome measurement were excluded. Narrative or systematic reviews with meta-analysis were considered for discussion and to retrieve unnoticed reports from the reference lists, but excluded from this review. Didactic records and letters to the author or editor were also excluded.

Considering the appraisal of the size of the HE, included reports had to be conducted in primary care, in outpatient clinics, or in healthy persons. Only published outcomes were considered and only primary outcomes were computed, without limitation. Included designs were RCTs, *post-hoc* analysis of RCTs, historical comparisons (pre–post comparisons), or observational studies. Studies conducted in hospital wards, in particular HH studies, were excluded.

The use of the term “Hawthorne effect” in health sciences is gradually increasing though its definition remains unclear. It is still more often used without any connection to the original studies in the Hawthorne plant, with a meaning of alteration of behavior related to an experimental background. In other disciplines, its meaning has mutated over time to become still more controversial (15). As our purpose was to investigate the HE in primary care research, we limited our investigations to medical research and our information sources to Medline and to the reference lists of the reviews. We hypothesized that the research in the reference lists of the reviews would provide any material that we would have missed by not exploring other sources. Besides this, PsycINFO and the Web of Science were searched to discuss the results.

The search used PubMed as the mean search engine. As McCambridge (15) and Purssell (17) did, we used the “Hawthorne effect” as the only keyword, though it is not a MeSH term (which is “effect modifier”). Filters were set for the availability of an abstract, for language (English, French), and for date range (2012-01-01 to 2022-03-31), as McCambridge’s last included report was published in January 2012. We deliberately chose not to use the keywords “observer effect*,” “participant reactivity,” or merely “reactivity” with another complementary term, in order to be consistent with McCambridge’s approach. The main difference with our search is that beside reports quantifying the HE, we also searched for reports giving a definition of the term. The terms “reactivity,” “placebo effect,” and “regression to the mean” were explored to discuss their interaction with the HE.

### Selection process

Initial selections of records were independently undertaken by two reviewers based on the availability of the record, the type of report, the title, and the abstract. All full-text reports meeting the inclusion criteria at this point were read. Reports retrieved from the reference lists of the papers and meeting the inclusion criteria were treated similarly. A consensus meeting of the two reviewers led to the final list of reports included in this review. All reports included were independently fully analyzed by the same two researchers.

### Synthesis methods and bias assessment

The same two researchers independently appraised the risk of bias and the level of evidence during the review of the selected full-text reports using the Cochrane tool (19).

Publication bias was assessed by a funnel plot using Review Manager 5.3^®^.

The narrative results regarding the definition of the HE have been summarized in Supplementary Table 1 with the description of the study, definition the authors used and a quality appraisal.

All published binary outcome measures of the mean outcome in studies conducted in primary healthcare, outpatient clinics, or healthy persons (e.g., students) have been included in a Microsoft Excel^®^ table. Studies included in the meta-analysis are summarized in Supplementary Table 2. Unpublished measures were not sought. Retrieved studies and measures were imported into Cochrane Review Manager 5.3^®^ to compute effect sizes and standard error. The generic inverse variance was used, adjusting for the direction of the HE (i.e., increase or decrease). The odds ratio (OR) and 95% confidence interval (95% CI) were computed using random effects in the context of an important difference in weight of the studies. Heterogeneity was computed using the *I*^2^ statistic. The result is presented as a forest plot. A supplementary sensitivity analysis was computed to differentiate odds ratios and heterogeneity by study design (Table 1) and by the level of evidence of the studies (Table 2) as the size of the HE appears to be associated with the quality of the research.

**TABLE 1:** Odds ratio and heterogeneity by study design.

Design	N	OR	95% CI	Chi^2^	df	P	I^2^ (%)
RCTs and pilot RCTs	6	1.08	[0.98; 1.19]	11.58	5	0.04	57
Quasi-experimental and *post-hoc*	4	1.19	[0.99; 1.44]	0.04	3	1 00	0
Observational	5	1.80	[1.22; 2.66]	126.32	4	<0.0001	97

N, number; OR, odds-ratio; 95% CI, 95% confidence interval; df, degrees of freedom.

**TABLE 2:** Odds ratio and heterogeneity by level of evidence.

Level of evidence	N	OR	95% CI	Chi^2^	df	P	I^2^ (%)
High/moderate	8	1.04	[0.99; 1.09]	8.02	7	0.33	13
Low	7	1.79	[1.27; 2.50]	128.67	6	<0.0001	95

N, number; OR, odds-ratio; 95% CI, 95% confidence interval; df, degrees of freedom.

### Ethics statement and reporting

No ethical statement is required in France for systematic reviews reusing already published data (research method classification MR-004).

The redaction of this review followed the Preferred Reporting Items for Systematic reviews and Meta-Analyses (PRISMA) statement update 2020 (20).

## Results

### Study selection

**FIGURE 1:**
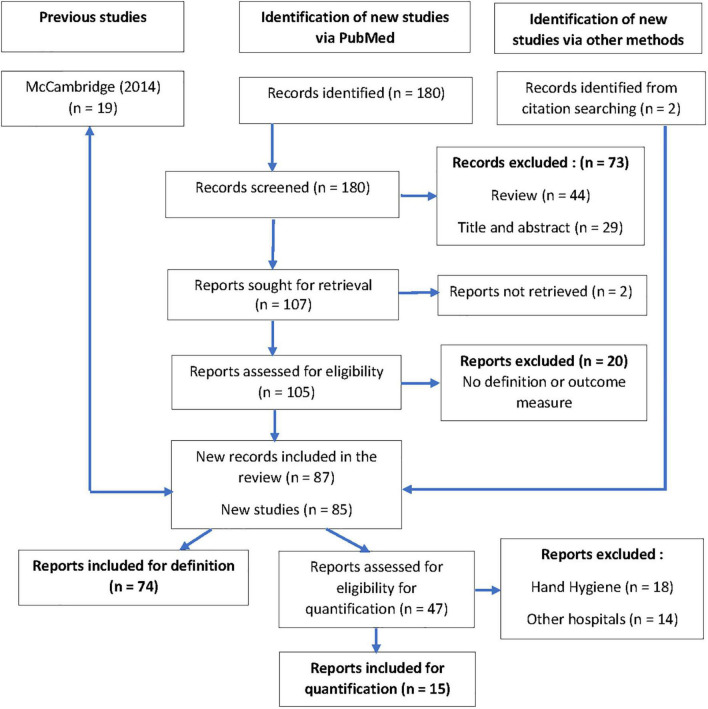
Flow diagram.

Of the 180 records found on Medline, two were excluded because of unavailable abstracts. Forty-four reviews provided two supplementary records from citation searching. Twenty-nine records were excluded based on title and abstract. Twenty reports were excluded after full reading because they cited the HE without definition or outcome measures. Twice two records reporting on the same study were included as they were complementary reports regarding the outcomes: Buckley (21), Ikpeze (22), Dal-Ré (23), and Pate (24). After the final selection, 74 new English-language reports were included and analyzed for definition and 15 for evaluation of the size of the HE in primary healthcare or outpatient clinics or healthy persons. No report in the French language was found (Figure 1).

### Study characteristics

Of the 74 selected reports in the definition branch, 15 were randomized controlled trials (RCTs) (25–39), two were not randomized controlled trials (40, 41), three were studies nested in RCTs (42–44), seven were retrospective reanalysis or discussions of RCTs (23, 24, 45–49), three were pilot studies prior to an RCT (50–52), and one was an RCT protocol (53). Further, there were 18 observational studies (54–71), 18 pre–post intervention studies or audits (21, 22, 72–87), one diagnostic accuracy study (88), four qualitative or mixed-method studies (89–92), one mixed-method study protocol (93), and finally one methodology protocol to build up research quality guidelines (13) (Supplementary Table 1).

Of the 15 purposely selected reports in primary care, outpatient clinics, or healthy subjects in the meta-analysis branch, the appraisal of the HE was based on a retrospective cohort pre–post intervention analysis in one study (72), in three studies on a *post-hoc* comparison of the RCT population to a non-RCT population (24, 30, 94), in three studies on the comparison of study parameters between enrollment and randomization in an RCT (28, 43, 51), in two studies on the comparison of persons consenting vs. not consenting to participate in a study (45, 67), in two studies on the follow-up of study populations exposed to repeated measurements (77, 95), and in four studies comparing a population being aware of exposure to observation or assessment to a population who were not aware (33, 64, 83, 96). The main binary outcomes that were inputted in the tables of the review manager to compute an effect size and standard error were sleeping time (28), anti-malarial drug prescriptions (33), time up and go measure (51), self-reported alcohol consumption (96), pain intensity (43), and subjective shared decision-making (95) in the RCTs or RCT feasibility studies. It was an antibiotic selection in a quasi-experimental RCT (30). In *post-hoc* analysis of RCTs, it was the influenza vaccination rate of students (94), acceptance of a video recording (45), and the rate of COPD acute exacerbations (24). In observational studies, we computed fall rates (72), protocol adherence (77), quality of care (64), school enrollment (67), and spontaneous eye blinks (83) (Supplementary Table 2).

### Risk of bias within studies

According to the Cochrane tool (19), in the definition branch, six studies had a low risk of bias (27–29, 31, 32, 39), 18 studies had a moderate risk of bias (24, 26, 30, 33–36, 38, 43–46, 48, 50, 52, 58, 69, 79), 38 had an important risk of bias (21, 22, 37, 40–42, 49, 51, 54, 56, 57, 59–68, 70–72, 74–78, 80–88), and two studies had a very important risk of bias (23, 73). Nine studies were not assessable with the tool (protocols or qualitative/mixed methods studies) (13, 25, 47, 53, 89–93).

In the meta-analysis branch, one study had a low risk of bias (28), seven a moderate risk (24, 30, 33, 43, 45, 94, 96), and seven a high risk (51, 64, 67, 72, 77, 83, 95).

### Results of individual studies

The included studies covered all five continents. The populations consisted of patients and various health professionals (students, nurses, physicians…) in different hospital wards or primary care and the community. The most commonly studied outcome was the World Health Organization (WHO) guidance for hand hygiene (HH) [“My Five Moments for Hand Hygiene” initiative (16)] in 13 studies (54, 56, 58, 60, 61, 65, 66, 71, 78, 79, 82, 89). It is noticeable that no study targeting this topic was conducted since the COVID-19 outbreak, except two qualitative ones (89, 92). Other outcomes were very heterogenous and linked to behavioral factors in health professionals and patients (e.g., completion of medical records, management protocol adherence, quality audits, antibiotic prescription, sleep duration, alcohol consumption) or other aspects (e.g., falls, skin infection, glomerular filtration rate, and glycemia).

### Results of syntheses

#### Definition of the Hawthorne effect in medical studies

Based on this review, our definition of the HE in medical studies is “an aware or unconscious complex behavioral change in a study environment, related to the interaction of four biases affecting the study subjects and investigators: selection bias, commitment and congruence bias, conformity and social desirability bias, and observation and measurement bias.”

##### A selection bias

The subject agreeing to participate in a study is interested in its outcome, expects a benefit, and trusts the investigator (67, 92). Characteristics of people who consent to participate in clinical trials often differ from patients who decline participation (24, 44). The investigator has a special interest in the field of the study, has more knowledge, and is more skilled in this field than the average health professional (45). As participants’ health literacy is essential to the ability to adhere to the study intervention as well as the ability to remember the details of the recommendations made to participants during visits, investigators will tend to include patients with a higher level of literacy (47).

##### A commitment and congruence bias

Signing the informed consent, the subject agrees to comply with the artificial experimental life rules and is willing to respect these rules as much as possible, far more than in real life (26). This is especially true for ambulatory active patients (like primary care patients) compared to passive inpatients (66). Signing his (or her) contract with the sponsor, the investigator agrees to follow good clinical practices, feels like part of a project, and has often agreed to undergo complementary training (77). In order to minimize the number of patients lost to follow-up, s/he will be particularly careful to strengthen the follow-up rules with the subject (47, 49, 59, 77).

##### A conformity and social desirability bias

As described by McCambridge, the “awareness of (…) having behavior assessed engenders [in the subject] beliefs about researcher expectations. Conformity and social desirability considerations then lead behavior to change in line with these expectations (15).” This is also true for the investigator: in case of uncertainty in the answers to an assessment scale, the investigator will tend to quote systematically in order to be in line with the expectations of the study that s/he shares (24, 50, 64).

##### An observation and measurement bias

The HE is often mitigated to the observation bias, without going more in depth into the concerns of this effect. The awareness of being possibly observed, assessed, and singled out engenders in the subject and in the investigator a special emphasis regarding the three previous biases (47, 58, 87). A direct observation (e.g., HH studies) engenders the largest HE (56) but depends on the authority status of the observer (65). If the observation remains distant, but the subject or the investigator has to complete repeated measurements or questionnaires, his/her interest in the field of the questionnaire will tend to change his/her behavior or beliefs (13, 24, 35, 95). This measurement bias is also described as “measurement reactivity” or “reactivity” (11, 13, 35, 97).

#### Heterogeneity of the Hawthorne effect

We found important differences across studies or within individual studies regarding the HE. Four main groups of factors seem to determine this heterogeneity: education and literacy or professional position, mental health conditions, environmental factors of the study setting, and the type of outcome measures.

##### The education or professional position of health professionals

There were important differences between nurses (more prone to HE) and physicians, and in physicians between medics (more prone to HE) and surgeons (14, 79). In subjects, the level of literacy and deprivation had an important influence with less marked HE in subjects with a lower level of education (66), though the embarrassment caused by the attendance of an observer might be higher in this population (57). Further, as already described, investigators tend to enroll in trial patients with a better health literacy as a means to ensure they understand and remember the recommendations made to participants during visits (47).

##### Mental health conditions modify the Hawthorne effect

The presence of symptoms such as anxiety and depression contribute to enhanced behavioral changes when people are aware of observation (45, 48, 70).

##### Environmental factors of the study setting

Regarding HH, the effect was clearly more marked in medicine wards than in surgery or anesthesia wards in hospitals (14, 79, 89). Primary care patients, playing an active role in the patient–doctor relationship, were more prone to the HE than more passive patients in a hospital setting. The HE was less pronounced in deprived dwellings, possibly increasing health inequalities (66).

##### The main outcome measure

The more the main outcome is linked to psychological or behavioral factors [e.g., sleep agendas (28) and alcohol consumption (38)], even when measured with blinded assessors, the more the effect is notable. The baseline level of the variable interferes also: the larger the deviation from the targeted value is at baseline, the more a HE has to be expected (71). However, as we will discuss below, this point has to be mitigated by a regression toward the mean (26, 43, 46). The direction of the targeted variation of the HE is also important: when the variable is expected to diminish [e.g., antibiotic prescription (52)], the relative reduction is more important than when it is expected to increase [e.g., carpal tunnel release (21, 22)].

#### Duration of the Hawthorne effect

The onset of the Hawthorne effect in a study environment is very fast (61). In HH studies, it was estimated to take 14 min after the appearance of the observer before health professionals altered their hand-washing behavior, increasing further after 50 min (71). In sleep agendas for sleeping trouble, there was a significant improvement in sleeping duration between the baseline measure and the measure at randomization; insulin resistance and fasting glucose improved simultaneously (28). In chronic kidney disease, there was an improvement in the glomerular filtration rate during the 3-month run-in phase of an RCT, in a disease where this usually worsens over time (50). In neck pain, the intensity of the pain diminished between screening and randomization (43).

The HE disappears totally or partially after the end of the observation or when the subject is released (36, 70, 85). In the case of long-lasting studies, the HE decreases gradually as the study environment becomes commonplace for the participants (33, 72, 87).

#### Size of the Hawthorne effect

As explained above, we only considered the appraisals of the effect on binary outcomes made in primary care research, outpatient clinics, and persons in good health (students) for the calculation of the size of the HE. Hand-hygiene studies were ruled out of our research since Purssell et al. published their meta-analysis (17). Our findings could only confirm theirs, and we consider these results as outdated as the COVID-19 outbreak considerably changed HH habits (18).

To compute the size of the HE, we purposely selected fifteen studies with different designs where the HE was appraised by different approaches (see study characteristics and Supplementary Table 2).

**FIGURE 2:**
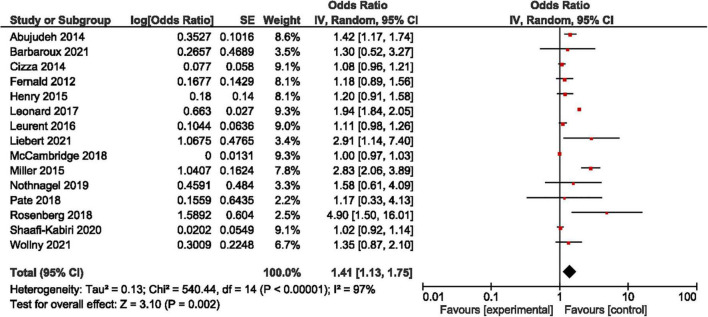
Size of the Hawthorne effect: Forest-plot of the meta-analysis.

We computed in all studies an OR of 1.41, 95% confidence interval [1.13; 1.75] (Figure 2: forest plot). In sensitivity analysis, we analyzed separately the studies by design (Table 1) and by the level of evidence (Table 2). It is notable that in RCTs, and in a quasi-experimental or *post-hoc* analysis of RCTs, the HE appeared to be not significant (95% CI respectively [0.98; 1.19] and [0.99; 1.44]) with a weak heterogeneity (*I*^2^ respectively 57 and 0%). The same observation is valid for studies with a high-to-moderate level of evidence (95% CI: [0.99; 1.09], *I*^2^: 13%). A significant HE with a high level of heterogeneity appears in observational studies and studies with a low level of evidence (95% CI respectively [1.22; 2.66] and [1.27; 2.50], and *I*^2^ respectively 97 and 95%).

### Reporting biases

Regarding heterogeneity in the meta-analysis of all the studies, it is notable that the *I*^2^ computing at 97% illustrates that the whole of the variance can be explained by heterogeneity. However, this heterogeneity is to be imputed to observational studies and studies with a poor methodology. Sensitivity analysis found that heterogeneity and the significance of the HE for binary outcomes disappear in well-designed controlled studies.

**FIGURE 3:**
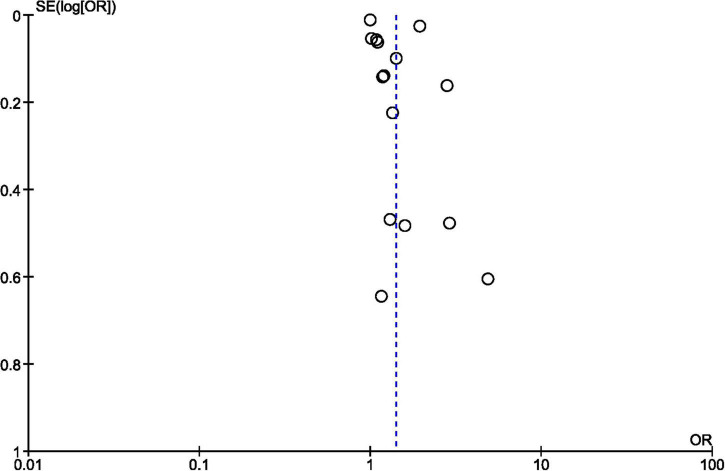
Funnel-plot of reports included in the meta-analysis.

Regarding the overall publication bias, the chimney plot did not illustrate an exaggerated risk with a well-balanced distribution of the results around the total OR (Figure 3: funnel plot).

## Discussion

### Summary of evidence

Researchers are still not unanimous regarding the existence of the HE and there is considerable inconsistency concerning the description and definition of the phenomenon (92). The point is not a denial of an experimental artifact which is unanimously agreed upon. The dissension relates to the description of what happened at the Hawthorne plant (10, 12). Rather than calling this artifact “participant reactivity,” we chose to keep the folkloric name of the Hawthorne effect as it is contemporarily used in health sciences, refining its definition. It is an experimental artifact that reduces the external validity and size effect of studies, with a combined OR for binary outcomes that can be carefully (due to heterogeneity) estimated at 1.41 (95% CI: [1.13; 1.75]) when considering studies conducted in outpatient clinics and with healthy persons. However, the significance and the heterogeneity of the HE are to be imputed to observational studies and studies with a poor level of evidence, as it disappears in well-designed RCTs or quasi-experimental studies. As a complex system of biases and psychological interferences, all related to a change of behavior in subjects and investigators, it is more dynamic than the summation of each individual bias.

The size and influence of the HE depend on the population being studied, the educational level and the social position of the investigators and subjects, the mental health status of the investigators and subjects, the studied variable, its initial value and its expected variation, and the duration of the experiment. It is possible to reduce this complex system by analyzing the behavioral beliefs and assessment of the issues of the intervention, the normative beliefs and motivation to comply, and the control beliefs and perceived power as described in the theory of planned behavior or reasoned action (98).

Up until recently, the HE has mainly been linked with observation bias, though the interaction between observation and selection bias has already been described (14, 67). To this point, the use of the term “Hawthorne effect” was of little interest as it was considered to be limited to the fact of observing a subject or an investigator in an experimental environment. The various publications of McCambridge have created a new association with social desirability bias and conformity bias (15, 99, 100). After having completed this review, we acknowledge the reality of what we chose to continue calling the Hawthorne effect, not only as an observation bias or as a summation of biases but also as a complex system that more or less creates an artifact in all research. Describing the HE as selection bias, commitment and congruence bias, conformity and social desirability bias, and observation and measurement bias is enlightening but somewhat simplistic as feedback loops are existing between the research targets, methods, and population explaining the important heterogeneity and temporal instability of the effect (101).

The HE must not be confused with other biases that are not related to bio-psychological, social, or behavioral factors, for example, attrition bias (102) or contamination bias (47). Furthermore, there are important overlap areas between the HE, the regression toward the mean (RTM), and the placebo effect. The RTM is a statistical phenomenon that occurs when repeated measurements are made on the same subject or unit of observation. It happens because values are observed with random error, that is a non-systematic variation in the observed values around a true mean (103). When patients are enrolled into a trial based on a deviating value of the main outcome and randomized a couple of weeks or months later, it can happen at randomization that the deviation of the main outcome is considerably reduced (26, 28, 43, 51). It is then difficult to differentiate the part of the HE and the one of the RTM. Regarding the placebo effect, similar to the HE, its definition is controversial which makes the distinction between the two effects difficult to exemplify. This effect is assumed to be caused by the special type of patient–provider interaction associated with giving and receiving a treatment, or in other words the treatment ritual (104). This patient–provider interaction can also be described without the prescription of any treatment, for instance, a patient who experiences pain reduction because of an interview with a warm and empathic physician (104). However, in this case the term of placebo effect, related exclusively to the medication, should not be used.

As a consequence, we can assume that all medical research, qualitative or quantitative, is inevitably prone to the HE which limits its external validity, starting with the conscious or unconscious selection of the study population and the investigators, leading to blind spots in medical knowledge.

### Strengths and limitations of this study

As an update of McCambridge’s review (15) and a continuation of Purssell et al.’s review (17), we chose to use but one keyword term: “Hawthorne effect.” Hence, we may have missed reports using as keywords the names of biases that are part of the HE (e.g., “observation bias” or “social desirability bias”) or alternative terms of the HE (e.g., “measurement reactivity” or “participant reactivity”). It is probable that our search strategy has been too specific, thus insufficiently sensitive. However, our choice was confirmed during the selection phase by the finding of reports using other terms appointing the same object or pointing to studies using these other terms.

The use of the term “Hawthorne effect” is widely used in medical sciences as we could note through the incrementally growing number of records citing it during the last 10 years in our search. It appeared to be relevant to refine the definition of the term as it is used contemporaneously in medical research in general and in primary care in particular. This is evident in 10 years after McCambridge’s review even though they had already noted a dissociation appearing in the meaning of the term in medical sciences in regard to other disciplines (15). For this reason, we only searched reports related to the medical field and we limited our search to Medline and the reference lists of the review articles that we retrieved. This choice might have been too specific and for this reason, we deepened our search using PsycINFO and the Web of Science in order to enlarge the consideration of the results in the discussion. The search in reference lists and other sources found, with two exceptions of reports that were considered in this review, records deriving from other disciplines, mainly from psychology and education sciences. It was notable that psychologists tended to use the term more in line with what happened at the Hawthorne plant and were more critical regarding its use, while medicals were more prone to use the term meaning an experimental artifact connected to behavioral changes in an experimental context, disregarding its origins. Considering the important number of reports that we analyzed and the definitions that were verified, the risk of having missed a definition due to a too-specific search seems minimal.

The limitation of our search to reports written in English and French might also have been detrimental. We missed two reports in Chinese about acupuncture, one in Japanese regarding HH, one in Dutch about drug effects, one in Spanish about the behavior of diabetic patients, and one in German about clinical coding. None of these reports gave a clear definition of the HE or could have been included in our meta-analysis. Further, the Dutch report might be confused between the HE and the placebo effect.

Some caution in the interpretation of the meta-analysis is necessary related to the fact that binary results (before–after or overt–covert comparisons) cannot exemplify a complex system. We note that adding “apples and oranges” may cause suspicion, but brought up less heterogeneity than HH studies using the same comparator in different hospital wards. This is related to the fact that the computed data for comparison in the meta-analysis are effect sizes and standard errors.

Considering the literature, this heterogeneity in the analysis of all studies was expected and we could have decided not to publish the computation of the meta-analysis as per Purssell et al. (17). In line with some authors, the sensitivity analysis confirmed the association between poor methods and the rise of a HE (11, 12). When analyzing separately RCTs and quasi-experimental studies, or studies with a good level of evidence, we noted that the presence of a HE in binary outcomes was no more significant with an acceptable heterogeneity. Rather, in observational studies or studies with a low level of evidence, the HE appeared to be significant, though with all of the variances possibly explained by heterogeneity.

### Implications of the results for future research

#### Randomized controlled trials

Randomized controlled trials (RCTs) in parallel groups are prone to the HE, but as groups are equally exposed to the effect, its impact on the main outcome might be reduced (99). This might be an explanation of the minor impact of the HE on binary outcomes. This is particularly true when the RCT is blinded, and if possible double blinded. However, blinded studies are often difficult or impossible to implement for ethical, practical, or financial reasons. Blinding would not prevent the selection of subjects to improve the homogeneity of the included population in order to enhance the chance of demonstrating statistically significant differences and reduce attrition bias or the occurrence of serious adverse events in a linear form of reasoning. Concomitantly, it would not prevent the selection of investigators with deeply rooted beliefs (like the role of cholesterol in leading to cardiovascular diseases) and a conformism that might be strengthened by complementary education, here again to improve homogeneity in completing the clinical record forms (CRF) (105).

Randomized controlled trials are often cluster randomized in primary care for feasibility reasons. The randomization level is mainly the GP investigator, and the cluster is defined as the group of patients of this GP. As a matter of fact, this emphasizes the influence of the selection of investigators on the results. The introduction of the intra-class correlation coefficient in the calculation (ICC) of the sample size is supposed to erase the effect of this bias on the results of the main outcome, but in most cases this ICC is estimated without certainty, based on the literature. Knowing the heterogeneity of the HE, the feasibility of computing exactly this ICC seems inaccessible.

The main risk, when the HE is not correctly mastered in an RCT, occurs when the effect size of the main outcome is small. If the size of the HE turns out to be important, it might overwhelm the results of the main outcome and lead to a negative trial (47). This is an important fact to consider when designing future RCTs in primary care or analyzing the events that led to a negative trial.

As noted, patients change their behavior by the start of the trial, and baseline values are prone to the RTM (24, 28, 43, 51). For these reasons, it can be recommended to separate enrollment in trials and randomization by about 1 month and to repeat outcome measures at the randomization visit. The analyzed baseline measures will be those at randomization, already modified by experimental artifacts, before the implementation of the intervention.

Implementing an RCT in primary care also means a profound disruption in the patient–doctor relationship. The latter changed during the past decades from a paternalistic model to a more balanced model of mutual participation (106). This relation can also be described by the family physician’s ongoing commitment to the patient and his/her family as persons (107). The physician will carefully choose among his/her patients, based on this mutual understanding, which patients s/he feels comfortable proposing participation in a trial to. This means that the physician who signed the study contract and the patient who signed the informed consent will both lose their freedom to share decision-making regarding a particular condition of the patient even in trials that try to avoid this barrier (108). In the PaCUDAHL-Gé trial (109), general practitioners had to propose to their insufficiently or unscreened for cervical cancer female patients home vaginal self-sampling or usual physician-sampled cervical smears. Patients included in the study could accept or refuse screening. The interest to include in the study all their eligible patients, whatever their decision, was repeated several times to the investigators by the study team. However, of the 300 included patients, 299 were screened (96 smears and 203 self-sampling) with only one who refused screening. It is also of note that no never-screened female patient was included. As cervical cancer screening is strongly associated with the level of health literacy, the preference of investigators to include patients with a higher level of literacy contributed to the exclusion of never-screened women (47).

Based on the findings of this review, we assessed whether the RCT we implemented regarding the impact of posters and pamphlets in GPs’ waiting rooms had been biased by a HE (2). The design of our study was a cluster-randomized trial, where GP investigators had no CRFs to complete as data were collected from a health insurance claim database. The GP investigators were not affected by the main outcome as it was the delivery of seasonal influenza vaccines in community pharmacies to patients targeted by this vaccination. The intervention was a reshuffle of the wall decoration of their waiting room, pre-existing posters and advertisements being taken away and replaced by one single poster promoting seasonal influenza vaccination, and the available reading material was removed and replaced by pamphlets of the same campaign. GP investigators gave their consent for this transformation without participating in it. GPs from the control group had their waiting room unchanged and had only to give their consent to access their data in the claim database. In this design, the only involvement of the GP investigators that might have biased the study was to give their consent to a study, where the vaccination coverage of their patients was assessed. This means (1) that they believed that seasonal influenza vaccination was important in their patients targeted for this vaccination and (2) that they were confident in doing their best to reach this objective. This means a selection bias of the GP investigators, but no observation bias (the observation of their outcomes being totally remote), no special commitment or congruence bias (their only commitment was signing the consent and accepting the reshuffle by others of their waiting rooms), and no special conformity or social desirability bias unless the one intertwined with the selection bias. It is thus that we believe that the HE in our study was minimal.

#### Observational studies

The HE probably has more consequences for the outcome of observational studies than RCTs, as it directly influences the results, without the balance of a control group. This statement matches the findings regarding observational studies in our meta-analysis.

The selection of the investigators in primary care will be influenced by the interest of the investigator in the topic and the prevalence of the studied condition among his/her patients. If patients are in general comparable, the way they are managed and educated by their physician might deeply differ due to a different level of commitment (i.e., for patients with addiction mainly managed by a small proportion of highly invested primary care physicians) (110). For similar reasons, the specialty of the physician can also lead to the selection of more complicated patients (e.g., diabetic patients or hypertensive patients managed by diabetologists or cardiologists are probably more difficult to balance and need heavier interventions than those managed by GPs though there is a lack of literature describing the difference in the burden of disease).

Observational studies will also ignore all the persons who are affected by a condition but are not aware of it or are not willing to address the condition. Similarly, it will ignore people who are not participating in diverse screenings. This highlights the problem of blind spots in primary care research.

Compared to usual care, conformity and social desirability will probably change the managing behavior of the investigator, the level of adherence and compliance of the patient, and the data collected in the CRF. Retrospective data will be altered also by conformity as well as by memory failure, with a trend to embellish vague recollections.

#### Qualitative research

Qualitative research collecting data rooted in semi-structured individual or group interviews will probably be biased by the HE when the interviewee is a patient or a doctor and the interviewer is a doctor him/herself. The relationship between a patient and a doctor or between two doctors will tend to increase social desirability bias and conformity bias because the interviewee is willing to meet the interviewer’s supposed expectations. This deviance might be even more underlined by the signing of a consent form and the recording of the interview that accentuates the need to provide an interest (111). As a criterion of reflexivity, a qualitative researcher is recommended to describe researcher characteristics that may have influenced the research, so including this HE (112).

Along the same lines, people who have a poor level of literacy or education will be more prone to refuse the interview as they are frightened they will not be able to reach the expected level of interest in the interviewer’s supposed expectations. Persons who feel guilty about breaking the rules in light of the norms of their social group (e.g., screening secretly for cervical cancer) will refuse the interview due to shame or fear of being discovered, or may not be willing to go further into transgression. In both cases, essential information will be lost to evidence.

## Conclusion

The Hawthorne effect results from a complex system of interacting psychological and social phenomena and appears in all experimental research thereby diminishing external validity. It combines the mobilization of feedback loops at different levels and time, encompassing social selection, individual motivation, commitment and congruence, social conformity and desirability, and the awareness of being observed, several times assessed, and singled out. There are overlapping areas with the regression toward the mean and the placebo effect. Observational studies or studies with a poor level of evidence are more prone to a HE.

## Data availability statement

The original contributions presented in this study are included in the article/Supplementary material, further inquiries can be directed to the corresponding author.

## Author contributions

CB designed the study, searched, selected, and analyzed the published reports, conducted the meta-analysis, and wrote the manuscript. OB searched, selected, and analyzed the reports and wrote the first draft. JF designed the study and tracked the conformance of data management. MC designed the study and amended the first draft. CC copyedited and revised critically the reports for important intellectual content. LP and PV supervised the design of the study and revised critically the manuscript for important intellectual content. All authors gave their final approval of the version to be published and agreed to be accountable for all aspects of the work in ensuring that questions related to the accuracy or integrity of any part of the work were appropriately investigated and resolved.
