# Supplementary material for: Defining and evaluating the Hawthorne effect in primary care, a systematic review and meta-analysis
Source: Front Med (Lausanne). 2022 Nov 8;9:1033486. doi: 10.3389/fmed.2022.1033486 (PMC9679018; doi:10.3389/fmed.2022.1033486)
Supplement: Supplementary file 1 [file Table_1.DOCX]

| **Table 1 : synthesis of reports defining the Hawthorne effect** | | | | | | | | | | | |
| --- | --- | --- | --- | --- | --- | --- | --- | --- | --- | --- | --- |
| # | Article | Study-characterisitic | Population | Setting | Field | Duration | Number of inclusions | Main Outcome | Results | Definition | Level of evidence |
| 1 | Abujudeh, 2014 [72] | Pre-post-intervention observational study | Department of radiology | Boston (MA) USA | Patients falls | 78 weeks | 327 falls in 5,080,512 radiology examinations | Fall rate, fall reports | An Increase, a plateau, and a decrease in incident reports | Awareness of being observed increasing reports, behaviour change in line with expectations and banalization with the time | Low |
| 2 | Afsarlar, 2016 [73] | Historic comparison | Male children in ER | Huston (TX) USA | Testicular torsion | 10 months | 28 controls,  29 intervention | Perioperative parameters | Improvement | Behaviour change in health professionals under observation | Very low |
| 3 | Ardestani, 2020 [42] | Study nested in a RCT | Post-stroke patients | Indianapolis (IN) USA | Gait analysis | ND | 15 | Spatiotemporal kinematics | Decrease of loading on the paretic limb unobserved | Observational awareness, differences between observed and unobserved conditions | Low |
| 4 | Arnold, 2020 [25] | Open label cluster RCT (protocol) | Nursing homes | Capital region of Denmark | Acute UTI | 10 months | 11 nursing homes and 637 residents | Antibiotic prescription for acute UTI | ND | a behavioral change with knowledge of trial participation | ND |
| 5 | Barron, 2022 [88] | Diagnostic accuracy study | Patients with compensated cirrhosis | Christchurch, NZL | Diabetes screening | ND | 20 | Screened diabetes | OGTT is the standard screening test for diabetes | participants were asked not to modify lifestyle behaviour during the study, some may have done so | Low |
| 6 | Blondeau, 2019 [26] | RCT | Patients with glaucoma non responding to latanoprost | Sherbrooke, Quebec, Canada | Ophthalmology | ND | 83 | Intraocular pressure | Reduction of intraocular pressure between enrolment and randomization | This selection bias can be affected by regression towards the mean and therefore may create a false clinical impression. When a patient enters a study, his compliance to the treatment can increase. | Moderate |
| 7 | Bhimani, 2016 [40] | Not randomized controlled trial | Rehabilitation ward nurses | St-Paul (MN) USA | Occupational health | 10 months | 62 nurses | Work-related musculoskeletal nursing injuries | 50% reduction in work-related musculoskeletal nursing injuries (not significant) | Nursing injury rates dropped before quality improvement interventions were put in place by self-engagement | Low |
| 8 | Briët, 2017 [27] | RCT  (cross-over) | Household | Ghana | Malaria prevention | 10 months | 83 households | Use of insecticidal nets | Fan use does not increase net use. Selection of households making a higher use of nets | Behaviour change in health professionals under observation | High |
| 9 | Buckley, 2013 [21] | Pre-post-intervention observational study | Arm, shoulder and hand surgery | Rochester (NY) USA | Carpal tunnel release | 8 months | Retrospective cohort: 39-patients. Prospective: 35 patients | DASH questionnaire | No difference between patients signing an informed consent or not | Alteration of the responses to a questionnaire resulting from the awareness of participation in a study | Low |
| 10 | Chandok, 2012 [74] | Pre-post-intervention observational study | Primary care | London (Ontario) Canada | hemochromatosis | 12 months + 60 months | ND | Genetic screening practices in non-study populations | Increase in HFE gene mutation testing, but constant proportion of patients with mutation | Improvement or modification of behaviour by a population as a consequence of it being affected by knowledge of studies | Low |
| 11 | Cizza, 2014 [28] | RCT | Obese outpatients | Bethesda (MD) USA | Sleep | 81 + 121 days | 125 subjects | Sleep parameters | Improvement between baseline and randomization | Behaviour and biochemical change in subjects under observation by the investigator; time dependent | High |
| 12 | Dal-Ré, 2018 [23] | Appraisal of a RCT: Salford Lung Study | COPD patients in primary care | Salford, UK | COPD | ND | ND | ND | ND | Behaviour change in investigator and patient under observation | Very low |
| 13 | Di Bona, 2020 [55] | Retrospective cohort study | Patients with severe asthma | Bari and Foggia, Italy | Asthma | 10 months | 15 consecutive patients | Patient-reported outcomes | improvements of all outcomes from baseline | a change in the behavior of an individual that results from their awareness of being observed | Very low |
| 14 | Edwards, 2013 [29] | RCT | Anaesthetists | Auckland NZL | Anaesthetics | 12 months | 400 medical records | Quality assessment grid | EMR better completed than handwritten records | Behaviour change in health professionals under observation and social desirability bias | High |
| 15 | El Saed, 2018 [56] | Observational cohort study | Healthcare workers | Riyadh, Saudi Arabia | Hand Hygiene | 10 months | 15,883 Hand hygiene opportunities | WHO guidance | Considerable overestimation of hand-hygiene compliance during overtly observation | Behaviour change due to awareness of being observed | Low |
| 16 | Fassett, 2014 [50] | Pilot study (before RCT) | Patients with chronic kidney disease | Brisbane, (Queensland) Australia | Chronic kidney disease | 27 months | 80 subjects out of the 132 included in the LORD RCT | estimated glomerular filtration rate | glomerular filtration rate improved during the 3 month run-in phase by 0.48 ± 2.90 ml/min/1.73 m²/month | Improvement or modification of study outcomes during the run-in phase of studies, modifying baseline outcomes. | Moderate |
| 17 | Fernald, 2012 [30] | Quasi-experimental RCT | Primary care physicians | Texas, North-Carolina  USA | Skin and soft tissue infections | 7 months | 91 family physicians (14 intervention, 77 control) | Antibiotic selection and prescription for abscesses | No difference between clinicians who participated in follow-up case reviews and 77 clinicians who did not | Study subjects' behaviour or study results are altered by the subjects' awareness that they are being studied or that they received additional attention | Moderate |
| 18 | Garrouste-Orgeas, 2012 [31] | RCT | Patients >18 admitted to intensive care | France | Intensive care | 12.5 months | 2117 patients | Prevention of medical errors | Efficacy to avoid errors for insulin administration and tube/catheter removal | Better performance of health professionals during study implementation disappearing after the end of the study | High |
| 19 | Goodwin, 2017 [57] | Observational study | Primary care patients and family physicians | Cleveland (OH) USA | Primary care encounters | 4 months | 138 family physicians in 84 practices | Effect of the observer on diverse criteria | Longer visit time, better history taking, structuring of the interaction and treatment planning | Behaviour change due to awareness of being observed and effect of the observer on the interaction in vulnerable patients | Low |
| 20 | Guerrero, 2013 [41] | Not randomized trial | Housekeepers | Cleveland (OH) USA | Infection control | 6 weeks | 117 sites | Disinfection of artificially infected surfaces | Improvement of disinfection of infected surfaces | Direct observation and real-time feedback | Low |
| 21 | Hagel, 2015 [58] | Observational study | Healthcare workers | Jena, Germany | Hand hygiene | 5 months | 8,158 Hand hygiene opportunities | WHO guidance | Strong positive correlation between directly observed compliance and electronically recorded | Tendency of people to behave differently when they know that they are being observed, including the psychological effect of being singled out, noticed, or made to feel important | Moderate |
| 22 | Hameed, 2017 [59] | Observational study | Family planning clients | Pakistan (70 districts) | Health service quality satisfaction | 2 months | 1,404 interviews at health facilities, 1403 at home | Service quality and satisfaction questions | Experiences reported in exit surveys at facilities were strongly biased positively for both experiential and perception-based questions | Health care providers pay more attention to their treatment and care of clients (“courtesy bias”)under surveyors’ observations | Low |
| 23 | Henry, 2015 [45] | Post-hoc analysis of a RCT | Depressive patients in primary care | San Francisco (CA) USA | Physician patient relation | ND | 135 investigators 867 subjects | Acceptance of video-recording | Selection in investigators and subjects. No change induced by video recording | Selection and behaviour change in health professionals under observation | Moderate |
| 24 | Humalda, 2020 [32] | RCT | Patients with chronic kidney disease | The Netherlands | Nephrology | 9 months (3 + 6) | 99 patients: 52 intervention vs. 47 control | Sodium excretion | Decrease during the intervention and increase during maintenance phase | Participants’ awareness of being in a sodium intervention study might have affected the outcome even without exposure to the intervention. | High |
| 25 | Ikpeze, 2018 [22] | Pre-post-intervention observational study | Arm, shoulder and hand surgery | Rochester (NY) USA | Carpal tunnel release | 8 months | Retrospective cohort: 39-patients. Prospective: 35 patients | QuickDASH questionnaire | Informed consent did not significantly alter patient responses to the QuickDASH questionnaire | Patients may alter responses to a questionnaire based upon the awareness of participation in a study | Low |
| 26 | Janssen, 2020 [53] | Cluster RCT  (protocol) | Mindfulness-based stress reduction | The Netherlands | Mental health in teachers | 12 months | 66 participants | Five Facet Mindfulness Questionnaire | ND | Participants’ awareness of being in a sodium intervention study might have affected the outcome even without exposure to the intervention. | ND |
| 27 | Kennedy, 2013 [75] | Pre-post-intervention observational study | Healthcare workers, Trauma and Orthopaedics | Cork, Ireland | quality control practice | 24 months (12 group A, 12 group B) | Group A: 105  Group B: 93 | Measurement of the tip apex distance in dynamic hip screws | The quality of operative fixation improved objectively following the institution of new form of outcome assessment | Psychological influence on individuals of being aware that their work is being examined, directly or indirectly, and the quality of their efforts consequently improving | Low |
| 28 | Kovacs-Litman, 2016 [60] | Observational study | Physicians vs. nurses, Hospital | Toronto, (Ontario), Canada | Hand hygiene | 2 months + 3 days | 4,906 Hand hygiene opportunities | WHO guidance | Covert observation produced much lower compliance than recorded by auditors. Difference in nurses >> physicians | Audits not only overstate performance overall, but can lead to inaccurate inferences about performance by professional groupings due to relative differences in the Hawthorne effect | Low |
| 29 | Kurtz, 2017 [61] | Observational study | Nurses in 5 intensive care units in 4 hospitals | Texas, USA | Hand Hygiene | 18 days | 65 nurses  3,620 Hand hygiene opportunities | WHO guidance | The presence of an observer did not significantly alter the behaviour of the nurses regarding their hand hygiene behaviour | A 20% difference in the hand hygiene adherence rate of the first 2 hours of observation and the last 6 hours of observation | Low |
| 30 | Laborie, 2022 [76] | Before and after observational study | Breastfeeding | Lyon, France | Breastfeeding | 24 months | 655 infants (301 before and 354 after) | Breastfeeding at discharge | significant improvement in both any breastfeeding and exclusive breastfeeding at discharge | Participation in medical research has been increasingly recognized to modify caregivers and patients’ behavior, regardless of the study design or intervention. Awareness of being observed which is related to a “social desirability consideration”. Improvement of caregiver behavior, technical knowledge, and their awareness of the importance given to breastfeeding. After an initial peak, the Hawthorne effect is known to diminish owing to habituation | Low |
| 31 | Lakomek, 2020 [62] | Prospective cohort study | Patients with out-of-hospital cardiac arrests | Osnabrück Germany | Cardiopulmonary resuscitation | 6 + 10 + 12 months | 292 patients: 95 + 94 - 103 | chest compression quality | The compression depth did not show increase after activation of sensor-feedback CPR | behavioural change due to an awareness of being observed | Low |
| 32 | Leonard, 2017 [77] | Pre-post-intervention observational study | Primary health care clinicians | Tanzania | Protocol adherence | Intervent.10 weeks  Assess.  18 month | 96 clinicians  4512 patients interviews | 4 measures of protocol adherence and 3 feedback visits | Being part of a project that encouraged quality, clinicians increased the quality of care in the short, medium and long-(18 months) term | When faced with the immediate attention and scrutiny inherent in any intervention, clinicians improve adherence but adherence falls as the attention diminishes | Low |
| 33 | Leurent, 2016 [33] | RCT | Healthcare workers | Tanzania | Management of anti-malarial drug prescriptions | 24 months | 19,579 patients in 18 facilities | Performance of a rapid diagnostic test and prescription of an anti-malarial drug | Improvement of the performance of tests and lower prescription of anti-malarial drugs in negative tests | Behaviour change in health professionals when their activity was overtly assessed (patients’ interviews). U-shaped association in time | Moderate |
| 34 | Liebert, 2021 [51] | Pilot study for a RCT | Patients with Parkinson’s disease | South Australia | Effectiveness of photobiomodulation to mitigate clinical signs of PD | 12 months | 12 participants: 6 immediately treated and 6 waitlisted | Time up and go measure of mobility | Improvement of time up and go measure between enrolment and treatment, and after 4 weeks of treatment | Occur in response to participation in research or being observed during a study. Appears to be transient, being short-lived during the treatment period and much diminished by 3 months. The waitlisted participants showed an improvement in outcome measures before treatment began | Low |
| 35 | Malchow, 2016 [63] | Deception observational study | Patients with a trans-femoral prosthesis for ≥ 6 months | Pittsburgh (PA), USA | Prosthetics and orthotics | 30 minutes | 3 patients | Gait cycle durations and several kinematic parameters | Users of lower limb prostheses appear affected by the presence of observers analysing their walking pattern | Psychological phenomenon under the umbrella concept of reactivity, stating that people will act differently when they are aware of being observed | Low |
| 36 | McDonald, 2018 [78] | Pre-post-intervention observational study | Healthcare workers in 2 medical wards | Montreal (Quebec) Canada | Hand Hygiene | 4 months (2 overt + 2 covert) | 418 Hand hygiene opportunities | WHO guidance | Dramatic increase of the compliance rate in overt compared to covert observation | Behaviour changes when the observed person is aware of being watched leading to a limitation or an obstacle to the accuracy of direct observation | Low |
| 37 | McDermott, 2016 [34] | RCT and cohort | health checks to identify risk of cardiovascular disease | Primary care, London, England | cardiovascular disease | 18 months | 12,459 participants | completion of the health check within 6 months of invitation | Uptake of a health check following an invitation letter is low and is not increased through an enhanced invitation method using the QBE. The offer of a £5 incentive did not increase the rate. | Larger effect sizes were observed for behaviours that the reviewers rated as easier to perform and more socially desirable. | Moderate |
| 38 | McKay, 2022 [89] | Qualitative study | Healthcare workers (nurses, registrars) | NSW, Australia | HH | 1 month | 3 nurses + 1 registrar? | acceptability of using video monitoring for hand hygiene auditing | Fears, concerns for patients, changes to feedback, | non-representative samples subject to the Hawthorne effect | ND |
| 39 | McLaws, 2018 [79] | Pre-post-intervention observational study | Healthcare workers in 2 wards | Sidney (NSW) Australia | Hand Hygiene | 24 months (2 x 3 audits) | Medical wards: 1,087,196  Surgical:  683,561 Hand hygiene opp. | WHO guidance | Increase of the compliance rate in overt *vs.* covert observation.  Medical wards > surgical.  No significant improvement by repeated audits | Epidemiologic errors associated with direct human auditing | Moderate |
| 40 | Miles, 2018 [13] | Methodology  (protocol to produce guidelines) | develop guidance on how to minimize bias in trials due to measurement reactivity | Manchester, UK | Measurement reactivity | ND | ND | To produce guidelines through an expert workshop | overall effects of asking questions on objective and subjective measures…there is considerable heterogeneity in effects…lack of pre-registration of protocols…publication bias | **Measurement reactivity** has been defined as being present where measurement in a research project results in changes in the people being measured. The changes can be behavioural, emotional or cognitive (e.g. beliefs)… There is also evidence that people taking part in research do so partly because they see personal benefit in doing so, including access to monitoring of their own health | ND |
| 41 | Miller, 2015 [64] | Observational study | Community health workers in primary care | Oromia region, Ethiopia | Childhood illness | 2 months | 137 health workers, 790 children | WHO Health Facility Survey tool (quality of care) | Differences between the two estimates relatively small for most of the indicators and borderline significant for only one indicator | Health workers perform better than under normal circumstances because they are being observed | Low |
| 42 | Morberg, 2018 [46] | Appraisal RCT | Patients with Parkinson’s disease | Odense (Denmark) | Efficiency of Transcranial Pulsed Electromagnetic Fields | ND | 95 participants | Unified Parkinson’s Disease Rating Scale (UPDRS) | Concerning the UPDRS, no treatment effect was found between the active group and the placebo group, albeit both groups improved | the Hawthorne effect should not be viewed upon as a single entity but rather as entities affecting outcome measures throughout the full study period. The Hawthorne effect should be seen as a potential cause of symptom alleviation in addition to the other causes likely to affect study outcomes, being the placebo effect, spontaneous remission, regression to the mean, selection bias, investigators measuring bias, participant reporting bias and fluctuation over very short time spans (hours) of PD symptoms.  Information meeting…already creating positive expectations of symptom alleviation... may also have changed behavior in us as investigators prior to study initiation | Moderate |
| 43 | Nair 2018 [35] | Open label RCT | Mothers with at least one child <5 years | Pune district in Maharashtra, India | Assessment of the TrackCare app | 6 months | 749 mothers (200 phone group, 100 control group, 449 cross-section control group (6 x 75)) | Childre seeking care | No difference in proportion of children seeking care | the participants are aware of their movements being monitored and repeated surveys (for validating recall) has the potential to alter health care seeking behaviour. The possible effect of altering a health-related behaviour as a result of exposure to a measurement device is called reactivity. | Moderate |
| 44 | Nothnagel, 2019 [43] | Feasibility study for RCT | Chronic neck pain patients | Jena, Germany | Assessment of pain intensity between enrolment and baseline | ND | 42 | Average pain intensity (VAS) | Reduction of pain intensity between enrolment and baseline | participants are more likely to enter a study when their pain is particularly intense… Regression to the mean is a purely statistical phenomenon, describing the general tendency for extreme values to converge towards a middle level… a person may change her or his behavior, experiences, emotions, etc., when becoming a study participant…interpreted as a type of reactivity to the situation, where a person is being systematically investigated and “observed”. A possible reason for this effect may be increased attention to factors that are related to the study outcomes… Thus, independent of the natural course of the disease, the inclusion procedure and the enrolment into a study may, in itself, have a major impact on the main study outcomes. | Moderate |
| 45 | Pan, 2013 [65] | Observational study | Healthcare workers by category | Taipei, Taiwan | Hand Hygiene | 12 months | 23,333 Hand hygiene opportunities | WHO guidance, 3 categories of observers | Compliance observed by medical students was significantly lower as than by infection control nurses and unit ambassadors | Performance usually improving when health care workers know that they are under observation, depending on professional category and category of observer | Low |
| 46 | Pate, 2018 [24] | Appraisal of a RCT: Salford Lung Study (SLS) | COPD patients in primary care | Stalford, UK | Management in Primary care of COPD | 12 months | Comparison of 1403 patients in the usual care arm to 16758 non trial matched patients in the Clinical Practice Research Datalink (CPRD) primary care database | rate of acute exacerbations of COPD | more exacerbations recorded in trial patients and behavioural changes in patients and general practitioner coding practices | the trial population may not be representative of the wider COPD population… participants or practitioners modify their behaviour due to an awareness of being observed... behavioural changes—for example, coding practices or number of COPD medications prescribed by GPs | Moderate |
| 47 | Persell, 2016 [52] | Test- Randomized controlled trial | Primary care physicians | Chicago (IL) USA | Management of antibiotic prescription | 24 months | 3,276 encounters before and, 3,099 during intervention | Appropriate and inappropriate antibiotic prescription | Large reduction in antibiotic prescription compared to the prior year regardless of the intervention or in controls | Behaviour change in primary care physicians in case of assessment. Possible floor effects in case of low baseline inappropriate prescription | Moderate |
| 48 | Petersen, 2021 [47] | Appraisal of two multicenter RCTs | Primary care patients with type II diabetes | Ulm, Germany | Management of type II diabetes | ND | ND | HbA1c | A limited effect size due to considerable improvements also in the control group | This effect is attributable to subjects’ knowledge of being part of a study, i.e., being observed and having data collected… Asking questions, for instance, induces rethinking about the current behavior… increased attention paid to the subjects by their HCPs… monitoring effort of clinical research associates… those control group patients that received a higher quality of standard care also showed larger improvements… it is expected that the majority of improvements induced by study effects occur between the first and the second data collection | ND |
| 49 | Petrini, 2021 [90] | Mixed methods (cross sectional survey + pair groups) | Health providers in an a Department of Anesthesiology and Critical Care Medicine | Philadelphia (PA) USA | Prevent burnout (human resources management) | ND | 58 physicians, 10 fellow physicians, 16 Certified Registered Nurse Anesthetist | Stanford Professional Fulfillment Index | identify important areas for improvement, build community, and target interventions to improve the well-being | deciding to call attention to a phenomenon may influence its manifestation… the act of observing influences an outcome | ND |
| 50 | Płaszewski, 2022 [93] | Mixed methods (web-based survey + focus groups) (study protocol) | registered physiotherapists located in Poland | Nationwide, Poland | Knowledge, skills, beliefs, and attitudes towards evidence based medicine | ND | About 1000 physiotherapists | In the cross-sectional survey: Evidence-Based Practice Profile Questionnaire | ND | people could change their behaviour or answer differently when being observed | ND |
| 51 | Quick, 2017 [66] | Observational study | Women in second pregnancy | Bradford, UK | Pregnancy health practices | 3 years | 316 (158 matched pairs) | 5 health behaviours and birth weight | Improvement in the number of women reporting any alcohol consumption. Estimates larger for women of higher education | The process of taking part in health research can improve participants’ health, independent of any intended intervention | Low |
| 52 | Rampersad, 2013 [80] | Pre-post-intervention observational study | Children in intensive care unit (anaesthetics) | Seattle (WA), USA | catheter associated blood stream infection | 12 weeks | 21 cases pre-intervention and 27 post-intervention | 3 appraisers’ assessments on video records (42 and 49 hours) | Changes in the clinical practices of the anaesthesia providers resulting in an increase in ‘clean’ behaviours | Tendency of providers to change their behaviour, trying to be compliant with whatever practice they thought was audited | Low |
| 53 | Rea, 2020 [91] | Qualitative study (Focus groups) | faculty and internal medicine residents in an outpatient clinic | Mayo Clinic, Rochester (MN) USA | Pedagogy: Perceptions of scheduled vs. unscheduled directly observed visits | ND | 14 faculty and 14 resident participants | Thematic analysis | Unscheduled observations were felt to be more authentic than scheduled observations and allowed for increased numbers of observations permitting more frequent formative assessments. Preference of remote video observation compared to in-room observation. | a change in behavior in response to observation and assessment… while learners are observed during patient care as their behavior may be consciously or subconsciously altered. | ND |
| 54 | Rezk, 2019 [81] | Observational pre-post study | Groin surgical site infections in vascular surgery | Jönköping County Hospital, Sweden | Antibiotic prophylaxis with Trimethoprim/ Sulfamethoxazole vs. Cloxacillin/ Cefotaxime | 3 years vs.2 years | 122 Cloxacillin/ Cefotaxime group vs. 67 Trimethoprim/ Sulfamethoxazole group | clinical examination and microbiological results, severity: Szilagyi classification | The change in antibiotic prophylaxis from Cloxacillin/ Cefotaxime to Trimethoprim/ Sulfamethoxazole was associated with an increased rate of inguinal SSI | The personal staff and operating vascular surgeons, in particular, in the study center was well aware of the problems with the high SSI rate, which have led to an individual change in behavior and a number of modifications of surgical technique, consciously or unconsciously, in order to reduce the SSI rate | Low |
| 55 | Rezk, 2021 [92] | Qualitative study (focus groups) | Health care professionals (surgeons, nurses, assistant nurses) in a vascular surgery department | Jönköping County Hospital, Sweden | how HCPs perceive being observed when following hygiene routines | ND | 44 health care professionals | qualitative inductive content analysis approach | Compliance is affected by many factors, not least a lack of communication between different groups of health care professionals | The HE is a type of observer effect, and is often cited as a source of bias in observed behavioural changes among study participants, or due to infection control interventions. There is considerable inconsistency concerning the description and definition of the phenomenon… the size and direction of the change in behaviour depend on the total time the participant is aware of being observed… it is a change in behaviour as a motivational response to the interest, care, or attention received through observation and assessment. | ND |
| 56 | Robles-García, 2015 [36] | Randomized controlled trial | Patients with Parkinson Disease | A Coruña, Spain | Neurology  Parkinson disease | ND | 15 Parkinson disease, 15 healthy | Gait pattern under overt and covert evaluation | Gait pattern modified under covert evaluation in both groups | Behaviour change in patients in an experimental environment even under covert observation | Moderate |
| 57 | Rosenberg, 2018 [67] | Observational study | Young women | Bush-buckridge (Mpuma-langa), South-Africa | HIV infection prevention | 48 months | 3889 young women | School enrolment | Cash transfers conditional on school enrolment did not influence HIV acquisition | Differences in school enrolment status were already apparent at the beginning of the study and grew larger as the trial progressed, diminishing the differences between study arms | Low |
| 58 | Sánchez-Carrillo, 2016 [82] | 3 phase pre-post-intervention observational study | Healthcare workers in a haemodialysis unit | Monterrey (Nuevo León) Mexico | Hand Hygiene | 4 months | 5403 Hand hygiene opportunities | WHO guidance | Direct observation might be inferior to video monitoring for evaluating hand hygiene compliance, related to bias in health worker and observer | Direct observation underestimates hand hygiene opportunities and health care workers are more compliant during direct observation study periods | Low |
| 59 | Shaafi Kabiri, 2020 [83] | Pre-post intervention | young healthy adult males with normal vision | Boston (MA), USA | Neuropathology | 7 minutes | 30 patients | Spontaneous eye-blink rate | transitory impact on blink count during the first and third minute of a passive image-viewing task that occurred immediately after subjects were informed of their eye blinks being counted | research participants who are aware they are being observed change their behavior, potentially biasing the outcome being measured | Low |
| 60 | Smith, 2015 [37] | Randomised controlled trial | patients in emergency departments with pain from traumatic injuries | Five hospitals in England | Pain control | 29 months | 200 patients | total pain experienced captured by visual analogue pain rating scale | Slightly (but not statistically significantly) lower total pain in the PCA group (mean difference 2.7, 95% confidence interval −2.4 to 7.8). | The nurses who were looking after the patients in the routine care group; they knew that they were being observed, so their review of the patients’ analgesic requirements may have been influenced by this. | Low |
| 61 | Smith, 2017 [38] | Randomized controlled trial | University students, usual drinkers | Sidney and Wollongong  NSW (Australia) | Alcohol addiction | ND | 114 regular drinkers | Alcohol use after 3 different brief alcohol interventions | Reduction of alcohol use whatever the intervention or without intervention | Effect of simple assessment requiring an important statistical power to show small effects of interventions | Moderate |
| 62 | Spector, 2012 [84] | Pre-post-intervention observational study | Healthcare workers | Bangalore (Karnataka)  India | childbirth-associated mortality | 6 months | 499 childbirth before intervention, 794 after | Use of the WHO Safe Childbirth Checklist program 29-item checklist | Marked increase in delivery of essential childbirth practices linked with improved maternal, foetal, and new-born outcomes | Subjects’ behaviour is influenced by an awareness of being observed; minimized by employing the same observers and observing in the same way in both phases | Low |
| 63 | Srigley, 2014 [68] | Observational study | Healthcare workers | Toronto (Ontario) Canada | Hand Hygiene | 14 weeks | 562 304 ABHR dispenses and 218 473 soap dispenses | WHO guidance | The hand hygiene event rate in soap and ABHR dispensers visible to auditors was significantly higher than in dispensers not visible | Observer bias, selection bias and tendency of people to change their behaviour when they are aware of an observer | Low |
| 64 | Steward, 2020 [69] | program implementation study (cohort study) | people living with HIV in Primary Care | Nationwide USA | Provision of HIV service delivery | 6 months | 14 demonstration project sites  15,738 clients | To Characterize the practice transformations that were ultimately implemented by the initiative’s demonstration projects and to examine the association of the transformations with retention in HIV care, ART prescription levels, and viral suppression | practice transformations are apotential strategy for addressing anticipated workforce challenges among those providing care to people living with HIV | clinical personnel who knew that they were under study were more motivated to make their projects a success | Medium |
| 65 | van Wyk, 2020 [44] | Nested study in RCT | Pregnant women with fetal growth restriction at term | The Netherlands | Obstetrics (comparison of labor induction with expectant monitoring)  Comparison of participants and non-participants | ND | 1116 women, 650 randomized, 466 declined randomization | 1) assessing whether and how baseline characteristics of nonparticipants differed from participants  2) comparing study outcomes of the 2 groups. | Nonparticipants in the DIGITAT have a worse outcome than the participants, despite the fact that these women were healthier at baseline. Most nonparticipants preferred expectant management and prolonged the possible undernourished fetal environment. Could explain the less favorable outcomes in these women | Characteristics of people who consent to participate in clinical trials often differ from patients who decline participation… socioeconomic status, and less educated women are often less willing to participate… participating in a clinical trial may have an effect on the behavior both of doctors and patients…adhere more strictly to protocols, perhaps leading to earlier or other interventions that could improve outcome. Patients may be more aware of risk factors because of the fact that they may be better informed regarding their condition owing to the study information provided before the trial. They may also feel that they are being watched | Medium |
| 66 | Vickers, 2017 [70] | Deception observational study | Individuals with and without chronic low back pain | Gainesville (FL), USA | Gait assessment | ND | 30 healthy persons, 25 with chronic low back pain | Gait assessment walking on a 8.4 m gait mat; Beck Depression Inventory (BDI) | The higher the BDI score, the greater the change in walking speed when overtly observed | Patients may alter their normal gait patterns when they are aware of observation. Symptoms of anxiety and depression contribute to gait modifications during observation | Low |
| 67 | Wainberg, 2020 [48] | Appraisal of a RCT | adult psychiatric patients | Brazil | HIV risk reduction | 4 years | 464 (233: increased information to HIV risk behaviors, enhance skills and motivation, 231: information about common chronic medical conditions) | Sexual Risk Behavior Assessment Schedule | HIV Prevention participants showed significant improvement in Information-Motivation-Behavioral domains: behavioral intentions were associated with significantly fewer unprotected sex occasions. Reduction of unprotected sex occasions was similar in controls. | asking patients detailed questions about their sex lives did not result in increased risk behavior, as has often been feared, and it might be speculated that repeatedly asking detailed questions about sex and risk might have contributed to risk-reduction in both intervention groups | Medium |
| 68 | Wander, 2014 [85] | Pre-per-post-intervention (DART trial) observational cohort study | Adults with non-traumatic out-of-hospital cardiac arrest | Seattle (WA) USA | Cardio-pulmonary resuscitation (CPR) | 156 months | 8,626 | Bystander CPR with or without dispatcher assistance | Compared to the before period, odds of dispatcher assisted CPR were higher during DART but no different after | The trial may influence dispatcher behaviour increasing arrest identification, provision of CPR instruction, and the proportion who received bystander CPR | Low |
| 69 | White, 2021 [86] | Pre-post intervention | none | Barts Health NHS Trust clinical transplantation laboratory (UK) | Improvement project identifying current Turnaround time performance and sources of delay. | ND | ND | Mean sample turnaround time and percentage completed within 5 days (KP2) | Performance on this control also increased comparably, but then fell away after our project finished, while it did not for B27/B57 | the improvement in TaT could be due to increased staff attention during the QI… the Hawthorne effect may have impacted performance during the PDSA cycles, but it is not responsible for (all) our new performance level for B27/B57. | Low |
| 70 | Wolff, 2014 [39] | Randomized controlled trial | Primary care patients | Cleveland (OH) USA | Medication reconciliation | 7 months | 367 patients, 20 family Physicians | Agreement between medication lists | Neither intervention improved medication lists agreement | Baseline agreement much higher than expected | High |
| 71 | Wong, 2020 [49] | Retrospective analysis of a RCT | Females with breast cancer in Ontario | Toronto (ON) Canada | Adjuvant breast cancer radiotherapy | 3 months | 346 patients declining participation in RCT compared to 349 patients enrolled in a RCT | compare the incidence of high-grade skin reactions | Lower pain score in the trial group compared to non-trial for conventional or hypofractionated radiotherapy. | closer follow-up, better patient adherence, more health aware patients have, or reaction to observation… known observation by researchers may alter behavior of participants due to awareness of being observed or having assumptions about the researcher expectations | Low |
| 72 | Wu, 2018 [54] | Prospective cohort observational study | Healthcare workers | Kaohsiung City, Taiwan | Hand Hygiene | 15 months | 31,522 hand hygiene opportunities | WHO guidance | Heterogeneity of the HE  Nurses > Physicians  Outpatients clinics > Intensive care units | Behaviour change in healthcare professionals under observation, heterogeneity depending on status and environmental factors | Low |
| 73 | Yin, 2014 [71] | Observational study | Healthcare workers | Multi-centre  USA | Hand Hygiene | 26 months | 11,444 hand hygiene opportunities | WHO guidance | 14 minutes for the appearance of the HE, increasing further after 50 minutes.  Depending on baseline complying rates and targeted improvement | Behaviour change in healthcare professionals under observation, heterogeneity depending on time and difference between baseline rates and targeted improvement | Low |
| 74 | Zhang-Rutledge, 2017 [87] | Pre-per-post-4 phases-intervention observational study | Pregnant women at childbirth | Houston (TX), USA | Episiotomy at childbirth | 60 months | 16,441 | Episiotomy rate | Reduction in the episiotomy rate without a reduction in the rate of operative vaginal delivery or an increase in the rate of third- and fourth-degree lacerations | Behaviour change under observation, disappearing gradually in time | Low |

**Keys:** **COPD**: Chronic obstructive pulmonary disease; **HH**: hand hygiene; **ND**: missing data; **NSW**: New South Wales; **NZL**: New Zealand; **RCT**: randomized controlled trial; **UTI:** urinary tract infection; **UK**: United Kingdom; **USA**: United States of America; **WHO**: World Health Organization
