# Supplementary material for: Defining and evaluating the Hawthorne effect in primary care, a systematic review and meta-analysis
Source: Front Med (Lausanne). 2022 Nov 8;9:1033486. doi: 10.3389/fmed.2022.1033486 (PMC9679018; doi:10.3389/fmed.2022.1033486)
Supplement: Supplementary file 2 [file Table_2.DOCX]

| **Table 2: synthesis of reports used to quantify the Hawthorne effect in primary care, outpatient clinics and healthy persons.** | | | | | | | | | | | |
| --- | --- | --- | --- | --- | --- | --- | --- | --- | --- | --- | --- |
| # | Article | Study-characterisitic | Population | Setting | Field | Duration | Number of inclusions | Main Outcome | Comparison | Results | Level of evidence |
| 1 | Abujudeh, 2014 [72] | Pre-post-intervention observational study | Department of radiology | Boston (MA) USA | Outpatients falls | 78 weeks | 327 falls in 5,080,512 radiology examinations | Fall rate, fall reports | Retrospective study comparing pre and post intervention time spans | An Increase, a plateau, and a decrease in incident reports | Low |
| 2 | Barbaroux 2021 [94] | Post-hoc analysis of a RCT | General practice residents | Nice, France | Influenza vaccination | 2 months | 161 healthy residents | Influenza vaccination | Control group of the RCT compared to a non-exposed group | No increase in vaccination uptake | Moderate |
| 3 | Cizza, 2014 [28] | RCT | Obese outpatients | Bethesda (MD) USA | Sleep extension | 81 + 121 days | 125 subjects | Sleep parameters | Comparison of parameters between inclusion and randomization | Improvement between inclusion and randomization | High |
| 4 | Fernald, 2012 [30] | Quasi-experimental RCT | Primary care physicians | Texas, North-Carolina  USA | Skin and soft tissue infections | 7 months | 91 family physicians (14 intervention, 77 control) | Antibiotic selection and prescription for abscesses | Randomly selected clinicians who participated in follow-up case reviews versus clinicians who did not | No difference between clinicians who participated in follow-up case reviews and 77 clinicians who did not | Moderate |
| 5 | Henry, 2015 [45] | Post-hoc analysis of a RCT | Depressive patients in primary care | San Francisco (CA) USA | Physician patient relation | ND | 135 investigators 867 subjects | Acceptance of video-recording | Clinicians consenting video-recording compared to clinicians non consenting | Selection in investigators and subjects. No change induced by video recording | Moderate |
| 6 | Leonard, 2017 [77] | Pre-post-intervention observational study | Primary health care clinicians | Tanzania | Protocol adherence | Intervent.10 weeks  Assess.  18 month | 96 clinicians  4512 patients interviews | 4 measures of protocol adherence and 3 feedback visits | Comparison of protocol adherence parameters in clinicians exposed to repeated measurements at 3 successive periods | Being part of a project that encouraged quality, clinicians increased the quality of care in the short, medium and long-(18 months) term | Low |
| 7 | Leurent, 2016 [33] | RCT | Healthcare workers | Tanzania | Management of anti-malarial drug prescriptions | 24 months | 19,579 patients in 18 facilities | Performance of a rapid diagnostic test and prescription of an anti-malarial drug | Comparison of days when exit surveys were conducted with other days | Improvement of the performance of tests and lower prescription of anti-malarial drugs in negative tests | Moderate |
| 8 | Liebert, 2021 [51] | Pilot study for a RCT | Outpatients with Parkinson’s disease | South Australia | Effectiveness of photobiomodulation to mitigate clinical signs of PD | 12 months | 12 participants: 6 immediately treated and 6 waitlisted | Time up and go measure of mobility | Comparison of selection and baseline data in waitlisted patients | Improvement of time up and go measure between enrolment and treatment, and after 4 weeks of treatment | Low |
| 9 | McCambridge 2018 [96] | 3 arm online RCT | Students | Newcastle (NSW) Australia | Alcohol consumption | 4 weeks | 4,583 students | Self-reported alcohol consumption | Students aware of an online survey compared to students aware that the survey is about alcohol consumption + AUDIT questionnaire | No evidence of any Hawthorne effect | Moderate |
| 10 | Miller, 2015 [64] | Observational study | Community health workers in primary care | Oromia region, Ethiopia | Childhood illness | 2 months | 137 health workers, 790 children | WHO Health Facility Survey tool (quality of care) | Comparison of register review data of children observed by the survey team during examination by community health workers and children not observed | Differences between the two estimates relatively small for most of the indicators and borderline significant for only one indicator | Low |
| 11 | Nothnagel, 2019 [43] | Feasibility study for RCT | Chronic neck pain patients | Jena, Germany | Assessment of pain intensity between enrolment and baseline | ND | 42 | Average pain intensity (VAS) | Comparison or neck pain intensity between enrolment and randomisation | Reduction of pain intensity between enrolment and baseline | Moderate |
| 12 | Pate, 2018 [24] | Appraisal of a RCT: Salford COPD trial | COPD patients in primary care | Stalford, UK | Management in Primary care of COPD | 12 months | Comparison of 1403 patients in the usual care arm to 16758 non trial matched patients in the Clinical Practice Research Datalink (CPRD) primary care database | rate of acute exacerbations of COPD | Comparison of COPD exacerbations in trial patients and in matched non-trial patients from the primary care CPRD database. | more exacerbations recorded in trial patients and behavioural changes in patients and general practitioner coding practices | Moderate |
| 13 | Rosenberg, 2018 [67] | Observational study | Young women | Bush-buckridge (Mpuma-langa), South-Africa | HIV infection prevention | 48 months | 3889 young women | School enrolment | Comparison of school enrolment of young women participant vs. non participant in a HIV prevention trial | Cash transfers conditional on school enrolment did not influence HIV acquisition | Low |
| 14 | Shaafi Kabiri, 2020 [83] | Pre-post intervention | young healthy adult males with normal vision | Boston (MA), USA | Neuropathology | 7 minutes | 30 patients | Spontaneous eye-blink rate | Comparison of the number of eye-blinks per minute in patients first not informed and after informed about the outcome measurement | transitory impact on blink count during the first and third minute of a passive image-viewing task that occurred immediately after subjects were informed of their eye blinks being counted | Low |
| 15 | Wollny, 2021 [95] | cluster RCT | Patients with poorly controlled type 2 diabetes mellitus | Rostock, Germany | Subjective shared decision making and patient centeredness in Primary care | 24 months | 833 patients 435 intervention, 398 control) and 108 GPs (54 intervention, 54 control) | effect of an educational intervention on the management of patients | Subjective shared decision making questionnaire between baseline and follow-up in both groups | Decrease of subjective shared decision associated to an increased patients’ demand for shared decision making and patient-centredness | Low |
